# Integrative analyses indicate an association between *ITIH3* polymorphisms with autism spectrum disorder

**DOI:** 10.1038/s41598-020-62189-3

**Published:** 2020-03-23

**Authors:** Xinyan Xie, Heng Meng, Hao Wu, Fang Hou, Yanlin Chen, Yu Zhou, Qi Xue, Jiajia Zhang, Jianhua Gong, Li Li, Ranran Song

**Affiliations:** 1 Department of Maternal and Child Health and MOE (Ministry of Education) Key Lab of Environment and Health, School of Public Health, Tongji Medical College, Huazhong University of Science and Technology, Wuhan, 430030 China; 2 Maternity and Children Health Care Hospital of Luohu District, Shenzhen, 518019 China; 3 Department of Epidemiology and Biostatistics, Arnold School of Public Health, University of South Carolina, Columbia, SC 29208 USA

**Keywords:** Genetic association study, Risk factors

## Abstract

It is challenge to pinpoint the functional variants among numerous genetic variants. Investigating the spatial dynamics of the human brain transcriptome for genes and exploring the expression quantitative trait loci data may provide the potential direction to identify the functional variants among autism spectrum disorders (ASD) patients. In order to explore the association of *ITIH3* with ASD, the present study included three components: identifying the spatial-temporal expression of *ITIH3* in the developing human brain using the expression data from the Allen Institute for Brain Science; examining the *cis*-acting regulatory effect of SNPs on the *ITIH3* expression using UK Brain Expression Consortium database; validating the effect of identified SNPs using a case-control study with samples of 602 cases and 604 controls. The public expression data showed that *ITIH3* may have a role in the development of human brain and suggested a *cis*-eQTL effect for rs2535629 and rs3617 on *ITIH3* in the hippocampus. Genetic analysis of the above two SNPs suggested that the over-dominant model of rs2535629 was significantly associated with decreased risk of ASD. Convergent lines of evidence supported *ITIH3* rs25352629 as a susceptibility variant for ASD.

## Introduction

Autism spectrum disorder (ASD) depicts a complex series of neurodevelopmental phenotypes resulting in a substantial burden for individuals, family and society^1–5^. Epidemiological surveys show that the prevalence of ASD is about 1 in 59 children aged 8 years in America^6^ and 1 in 100 children aged 6–10 years in Jilin, a city in northern China^7^. The key part of ASD susceptibility is estimated to be caused by common variants^8^.

The application of genome-wide association studies (GWAS) in recent years has made rapid progress in the identification of genes whose variants significantly increase the susceptibility of ASD^9–14^. However, due to the issue of false-positives that results from massive number of statistical tests in GWAS^15,16^, many ASD-risk genetic variants were not successfully validated across different samples^17–19^. With the available public datasets, it is now possible to investigate the spatial dynamics of the human brain transcriptome for candidate genes described in previous GWAS and incorporate the expression quantitative trait loci (eQTL) derived from brain tissues, which may provide the potential direction to identify the functional variants. Such comprehensive researches have made achievements in many kinds of psychiatric disorders^20^, schizophrenia(SCZ)^21,22^, attention-deficit hyperactivity disorder(ADHD)^23^, and bipolar disorder(BP)^24^ to name a few.

Among the ASD susceptibility genes (*TRIM33*, *CDH9*, *CNTN4*, etc.)^9,10,13,14,25,26^ found by GWAS, *ITIH3* (inter-alpha-trypsin inhibitor heavy chain 3, containing 24 exons and spanning 14.2 kb in the genome, located in 3p21.1) was the suspicious risk gene for ASD. The single nucleotide polymorphism (SNP) rs2535629 in *ITIH3* was associated with the combination of five traits, including ASD, ADHD, BP, SCZ and major depressive disorder(MDD)^26^. The *ITIH3* rs3617 was associated with the combined ASD and schizophrenia analysis^14^ and ASD^27^ in GWAS. On the other hand, these SNPs didn’t achieve genome-wide significance in other ASD GWAS^9,10,25^. Nevertheless, the association between rs2535629 and rs3617 and antipsychotic response and other psychiatric disorder was in favor of their potential influence on the risk of ASD^28,29^. Moreover, a past study discovered that the *Ambp/bikunin* (necessary for functional *ITIH1* and *ITIH3* complexes) knockout mice exhibited increased anxiety‐like behavior, reduced exploratory activity and alterations in social approach^30^. These evidence suggested a controversial association between *ITIH3* and ASD susceptibility.

Thus, in our study, in order to explore the association of *ITIH3* with ASD, we first performed the spatio-temporal expression analysis of *ITIH3* using the data from Brainspan. Second, we examined the *cis*-acting regulatory effect of rs2535629 and rs3617 on the *ITIH3* expression using UKBEC database^31^. Last, to further investigate whether rs2535629 and rs3617 confer risk of ASD, we conducted a genotyping experiment in a Chinese Han sample of 602 ASD cases and 604 controls.

## Results

### Spatio-temporal expression pattern analysis of *ITIH3*

**Figure 1:**
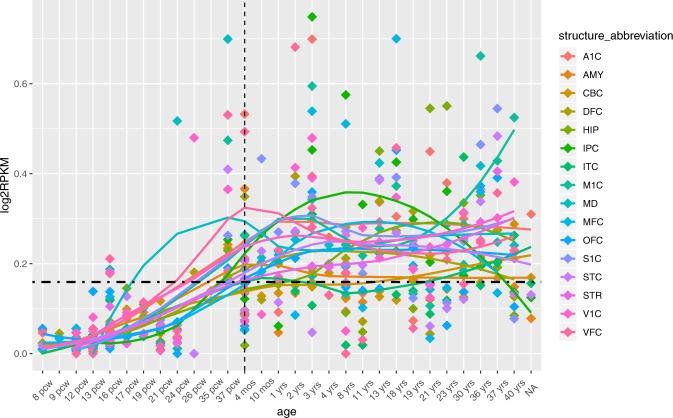
*ITIH3* expression in brain subtissues across brain development (with RNA-seq RPKM values from the BrainSpan Atlas v.10; shown from 8 postconception weeks (pcw) to 40 years of age (yrs)). Birth is indicated by a vertical gray dashed line, and the mean expression across all time points is indicated by a horizontal black dashed line. The Y-axis is log_2_ (RPKM values). The brain structures are primary auditory cortex (core) (A1C), amygdaloid complex (AMY), cerebellar cortex (CBC), dorsolateral prefrontal cortex (DFC), hippocampus (HIP), posteroventral (inferior) parietal cortex (IPC), inferolateral temporal cortex (area TEv, area 20) (ITC), primary motor cortex (area M1, area 4) (M1C), mediodorsal nucleus of thalamus (MD), anterior (rostral) cingulate (medial prefrontal) cortex (MFC), orbital frontal cortex (OFC), primary somatosensory cortex (area S1, areas 3,1,2) (S1C), posterior (caudal) superior temporal cortex (area 22c) (STC), striatum (STR), primary visual cortex (striate cortex, area V1/17) (V1C), and ventrolateral prefrontal cortex (VFC).

As shown in Fig. 1, the expression of *ITIH3* was lowest before 12 postconception weeks and rose little by little until birth. Later, the high-level expression was retained to adulthood. The permutation test showed that the expression pattern was not caused by chance (*P* < 0.001). The fluctuation with brain development in developing human brain indicated that *ITIH*3 may be involved in brain development. The specific data was shown in supplementary material.

### Association of rs2535629 and rs3617 with higher expression level of *ITIH3* in hippocampus

**Figure 2:**
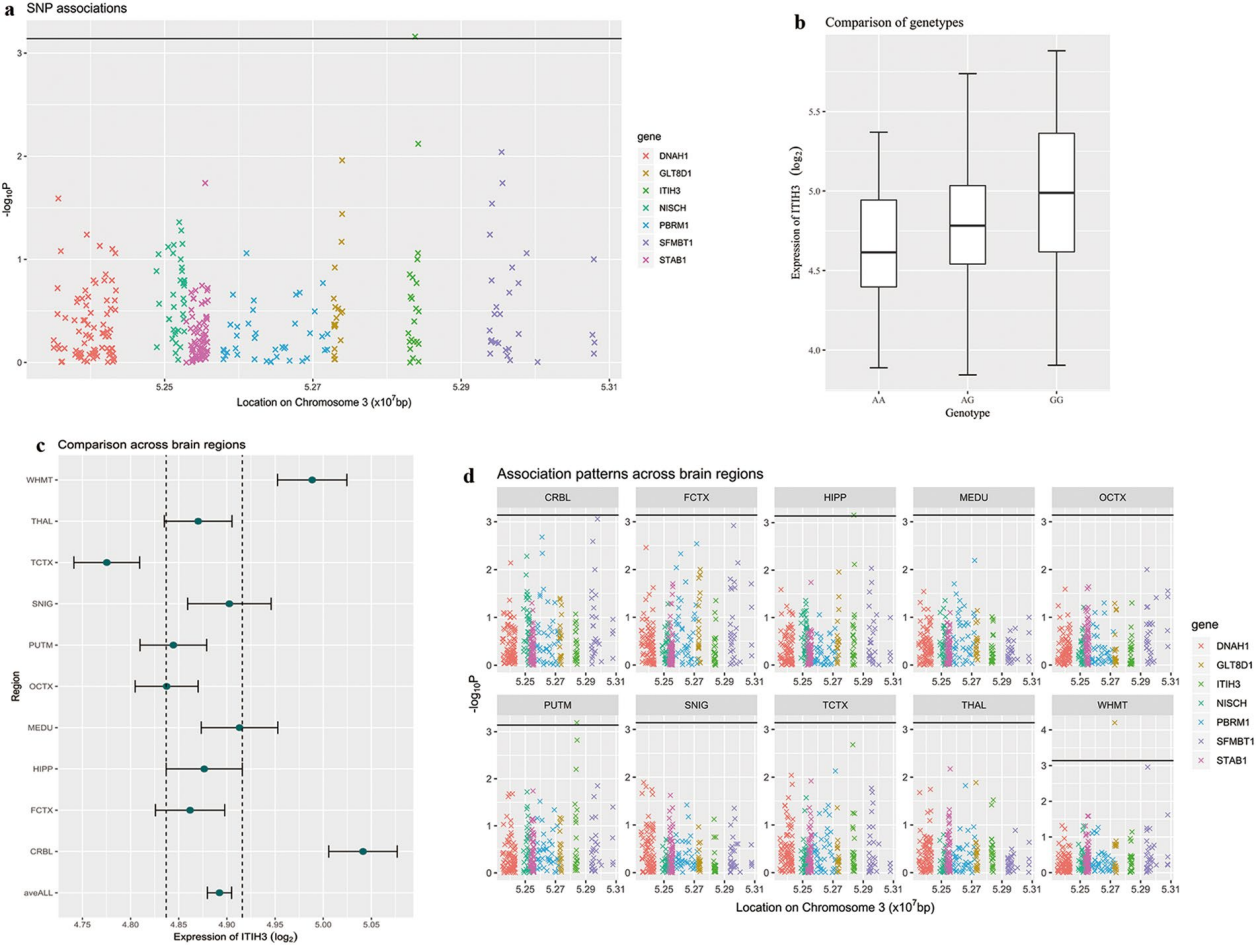
Association of rs2535629 and expression level of *ITIH3* in hippocampus(HIPP). (**a**) Significance level of associations between rs2535629 and gene expression levels of nearby genes of *ITIH3* (the probes used by Affymetrix were organized according to locations of their starting base at chromosome 3). **a** was a zoom-in view of (**d**) “HIPP”. **b** Comparison between gene expression levels of *ITIH3* at hippocampus based on microarray expression data with different genotypes at SNP rs2535629. (**c**) Comparison on gene expression levels (mean value and 95% confidence interval) of *ITIH3* across 10 brain regions, including inferior olivary nucleus (MEDU; subdissected from the medulla), putamen (PUTM; at the level of the anterior commissure), substantia nigra (SNIG), cerebellar cortex (CRBL), thalamus (THAL; at the level of the lateral geniculate nucleus), temporal cortex (TCTX), intralobular white matter (WHMT), occipital cortex (OCTX), frontal cortex (FCTX), and hippocampus (HIPP). (**d**) Association patterns between SNP rs2535629 and gene expressions in 10 brain regions. Genes with significant associations (*P* < 0.0007, calculated by 0.05/10/7 by Bonferroni correction) were above the black line. bp Indicates base pairs.

For *ITIH3* rs2535639, there was 16 AA, 53 AG, and 64 GG in the 134 samples in the brain eQTL database from the UKBEC. The data showed that the carriers of rs2535629 G allele displayed higher expression of *ITIH3* (*P* = 0.00069) in the hippocampus (Fig. 2a,b). In addition, although *ITIH3* was expressed widely in the brain (Fig. 2c), this eQTL association was particular for the hippocampus and putamen and was not indicated in the rest of brain regions (*P* < 0.0007, Bonferroni correction for 7 neighboring genes and 10 types of brain tissues) (Fig. 2d). Except for the *ITIH3* gene, rs2535629 was also associated with higher expressions of *GLT8D1* in the intralobular white matter (WHMT) (*P* = 0.00063).

**Figure 3:**
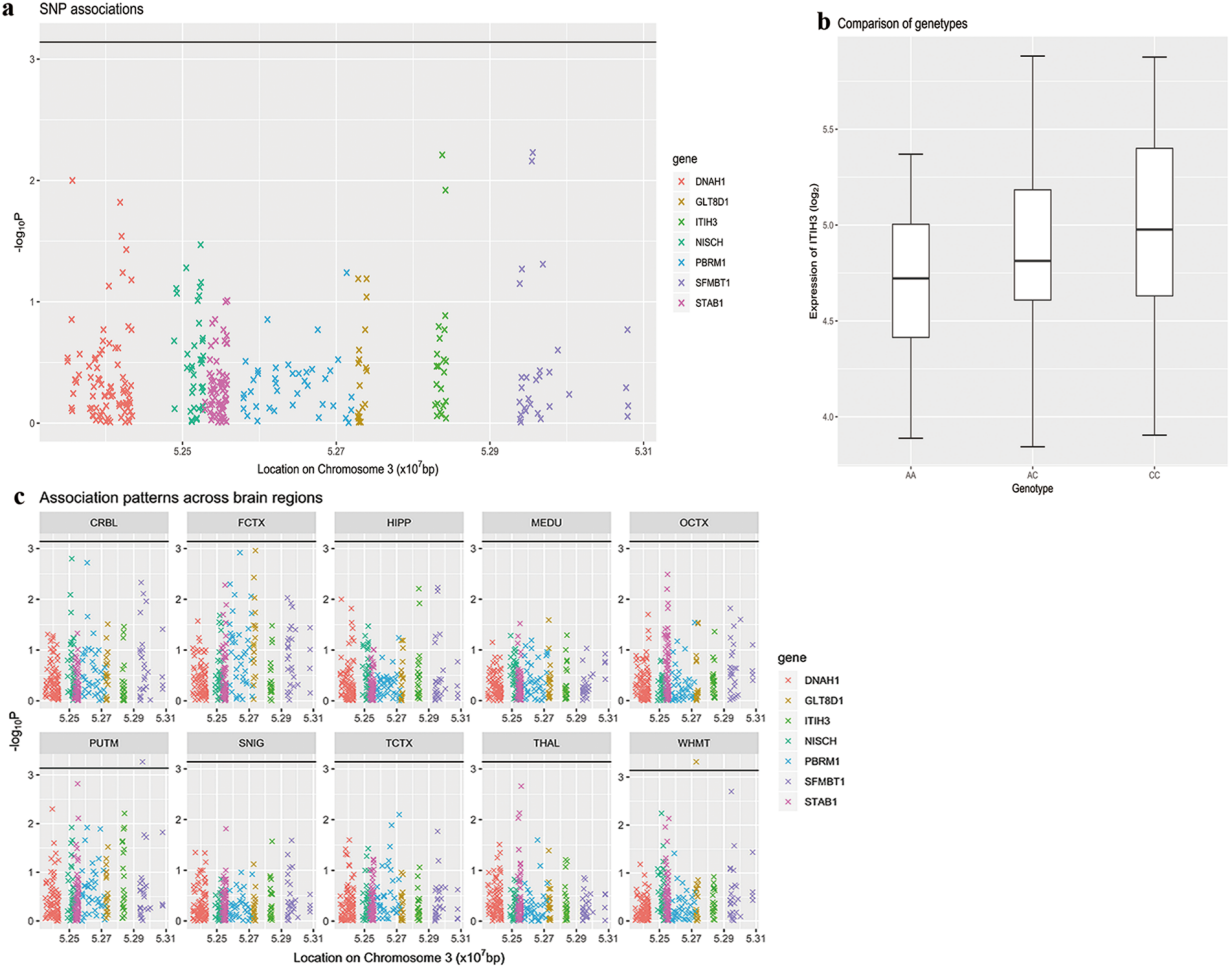
Association of rs3617 and expression level of *ITIH3* in hippocampus. (**a**) Significance level of associations between rs3617 and gene expression levels of nearby genes of *ITIH3* (the probes used by Affymetrix were organized according to locations of their starting base at chromosome 3). **a** was a zoom-in view of **c** “HIPP”. (**b**) Comparison between gene expression levels of *ITIH3* at hippocampus with different genotypes at SNP rs3617. (**c**) Association patterns between SNP rs3617 and gene expressions in 10 brain regions. Genes with significant associations (*P* < 0.0007, calculated by 0.05/10/7 by Bonferroni correction) were above the black line. bp Indicates base pairs.

As for *ITIH3* rs3617, there was 26 AA genotypes, 62 AC genotypes, and 46 CC in the samples from the UKBEC. The carriers of rs3617 C allele displayed higher expression of *ITIH3* (*P* = 0.0062) in the hippocampus (Fig. 3b). This eQTL association was lost for the hippocampus and the rest of brain regions after Bonferroni correction for 7 neighboring genes and 10 types of brain tissues (Fig. 3a,c). In addition to gene *ITIH3*, rs3617 was also associated with lower expressions of *SFMBT1* in the putamen (*P* = 0.00054) and higher gene expression of *GLT8D1* in the WHMT (*P* = 0.00048). The role of rs2535629 and rs3617 in regulating their target genes in the brain tissues were also supported by evidence from GTEx database. For rs2535629, the 3DIV database showed evidence of chromatin interactions in human hippocampus, which further supported it as an eQTL (Fig. S1). We also explored the spatio-temporal expression pattern analysis of the other six SNP *cis*-regulatory effect genes, including *DNAH1*, *GLT8D1*, *NISCH*, *PBRM1*, *SFMBT1* and *STAB1* genes (Fig. S2). The mRNA expression levels of these genes were higher/lower at postconception stage compared with the later stages (permutation test *P* < 0.001). The specific data was shown in supplementary material.

### Association of rs2535629 and rs3617 with ASD in the Chinese Han population

#### Subjects’ characteristics

In the case-control study, 602 ASD patients (520 males and 82 females, 5.39 ± 2.431 years) and 604 healthy controls (515 males and 89 females, 6.24 ± 1.930 years) were recruited. The ratio of boy to girl between cases and controls was 6 to 1, and the two groups were matched by gender (*P* = 0.579).

#### Association analysis between individual SNPs and ASD risk

**Figure 4:**
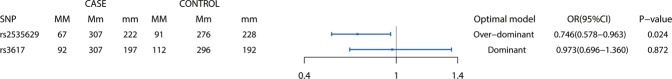
Association between SNPs and ASD. There are three genotypes, including homozygote of major allele (MM), heterozygote (Mm), homozygote of minor allele (mm).

The two SNPs followed the Hardy-Weinberg equilibrium (*P* > 0.05). The statistical power for detecting the effects of the SNPs were 93.6% and 94.0% (Supplementary Table S1). As shown in Fig. 4, the over-dominant model of *ITIH3* rs2535629 was significantly associated with decreased risk of ASD (OR = 0.746, 95% CI = 0.578–0.963, *P* = 0.024). The rs3617 polymorphism didn’t show the significant association with the risk of ASD. The specific data was shown in supplementary Table S2.

## Discussion

In this study, we applied the spatio-temporal expression analysis to the *ITIH3* gene. The expression level of *ITIH3* was relatively low at the early developmental stages; as development progresses, the expression of *ITIH3* gradually increased in human brain. The fluctuation of *ITIH3* expression indicated that *ITIH3* may be involved in brain development^21^. We then examined colocalization of the SNPs with eQTLs. Rs2535629 had a *cis*-acting regulatory effect on the gene expressions of *ITIH3* in the hippocampus, as was the polymorphism rs3617 before the Bonferroni correction for 10 types of brain tissues and 7 neighboring genes. The two SNPs had further eQTL evidence in brain tissue from GTEx and 3DIV database. Based on the above results, we carried out a case-control analysis to examine their associations with ASD. The genotyping analysis results suggested that rs2535629 was associated with ASD in the Chinese Han population. Integrating the human brain transcriptome and brain eQTLs appeared to be a useful approach to identify functional variants in this study before validating the previous GWAS results in independent samples.

*ITIH3* showed genome-wide significant association with neurodevelopmental disorders in European populations. The GWAS study consisting of 33332 cases and 27888 controls for five disorders including ASD, ADHD, BP, MDD and SCZ, found that rs2535629 in *ITIH3* was significantly associated with the combination of the five traits (*P* = 2.54 × 10^−12^, OR = 1.10)^26^. Meta-analysis of the combination of Psychiatric Genomics Consortium (PGC) ASD GWAS data with the PGC schizophrenia GWAS found the significant association between *ITIH3* rs3617 with ASD and schizophrenia (*P* = 3 × 10^−12^, OR = 1.08)^14^.

It had indicated that rs2535629 is relevant in antipsychotic response in patients of European ancestry^29^. And the expression level of *GLT8D1* was positively correlated with the number of G alleles of rs2535629 in the whole blood samples of major depressive disorder (MDD) in Japanese subjects^32^. Similar result was found in the WHMT in the present study. Rs2535629 has a RegulomeDB score of 1b, suggesting that it might be functional. Considering that the carrier of allele G at rs2535629 was responsible for higher expression of *ITIH3* in hippocampus using UKBEC database and allele A was risk allele rather than G allele in the case-control study in Chinese Han population, we ventured that the decreased expression of *ITIH3* may contribute to the risk of ASD. However, in the family-based association study of 640 Chinese Han autism trios, rs2535629 was not a susceptibility variant for autism^33^, which was not consistent with our findings. This disparity may due to that China has a vast territory and the population’s ethnic composition is very complicated. Wang’s study recruited participants from Beijing, located in the north of China. Our participants were mainly from the southern Chinese city of Shenzhen. In addition, our study compensated for the non-randomness of the family study design, as the data collected by this family design was only confirmed by certain cases, leading to the existence of confirmation bias^34^. The precise function of rs2535629 is unknown, as it is in the intron region of *ITIH3*. More studies, such as in-vireo reporter system (i.e., pGL3 Luciferase Reporter Vectors) are needed to validate the association between rs2535629 and ASD and the potential mechanisms.

As for the rs3617, at the time of writing, there are little to no completed case-control or family-based ASD studies. One study in Spain found that rs3617 could not confer a risk of developing schizophrenia (SZ) and bipolar disorder (BD) in individuals who were already at high risk due to their family history^35^. This result was partially agreement with our finding. Further analyses based on substantially larger sample size are required to further investigate the role of rs3617 in ASD.

The synaptic strength in the hippocampus is responsible for the memory formation and consolidation^36,37^. The deficits in memory consolidation are associated with ASD. The offspring of mouse maternal immune activation (MIA) model exhibited different feature distributions of hippocampus sharp-wave ripples (SWRs) waveforms^38^. The results of functional magnetic resonance imaging (fMRI) showed that the ASD cases exhibited reduced hippocampal connectivity compared to control group^39^. These results indicated that the rs2535629 might impact the expression of *ITIH3* in the hippocampus and then contribute to the pathogenesis of ASD. Neurobiological studies from this perspective are necessary.

The limitation of this study was that we did not exactly match our controls to cases according age. The study was based on the hypothesis that human genotypes generally do not change with age. Besides, the BrainSpan and UKBEC database used for expression analysis comprise samples mostly from UK and US, rather than China. Currently the publicly databases comprising the gene expression data from Chinese are not available.

In summary, this study presented a series of convergent lines of evidence that support *ITIH3* rs25352629 as a susceptibility variant for autism in the Chinese Han population. The present study provides evidence of feasibility of exploiting the information using public repositories which may provide a direction for GWAS analysis in the independent samples. This issue that we did successfully find the ASD susceptibility variant in the Chinese Han population on the basis of the combination of public database, highlight the informative value of integrating brain expression and eQTL data in advancing our use of GWAS results. Such approach could be further investigated in similar studies in the future.

## Methods

### Spatio-temporal expression pattern analysis of *ITIH3*

ASD is a neurodevelopmental disorder. Recent studies have shown that ASD risk genes, such as neurexins, played one part in the brain development through altered the function of synapses^40,41^. If *ITIH3* gene is involved in brain development, it may be expressed in developing the human brain. We downloaded the expression data from the BrainSpan: Atlas of the Developing Human Brain^42^ (http://www.brainspan.org/rnaseq/search/index.html) (access date: 10/16/2016) (n = 42 individuals). The expression data includes 31 developmental time points (from 8 post-conceptional weeks (pcw) to 40 years (yrs)) and 26 brain regions. We excluded 10 brain regions where the number of *ITIH3* gene expression was less than 5. The expression data is RNA sequencing (RNA-Seq) and standardized by RPKM. We used the log_2_ (RPKM values) for the analysis. The permutation test was used to evaluate whether the observed expression pattern is expected by chance or not.

### Association of rs2535629 and rs3617 with expression level of *ITIH3*

We used the UKBEC database^31^ to explore the *cis*-acting regulatory effect of SNPs on the effect of *ITIH3*. Gene expression data are available for 10 brain regions from 134 neuropathologically free participants. For the rs2535629 and rs3617, we tested whether the SNP was associated with expression of this gene. Then we went on to test whether such an association was tissue specific and whether this SNP had *cis*-regulations on expressions of the nearby genes. For this extended exploration, we corrected for multiple comparisons between the number of nearby genes and the number of brain areas^22^. Considering the limited sample size in the UKBEC database, we also explored the Genotype Tissue Expression project (GTEx V8, including 17,382 RNA-Seq samples from 948 donors)^43,44^. In addition, we searched evidence of chromatin interactions in hippocampus tissues available from 3DIV—a 3D-genome interaction viewer and database^45^.

### Association of rs2535629 and rs3617 with ASD in the Chinese Han population

#### Ethical approval and informed consent

The experiments of the article were approved by the Ethics Committee of Tongji Medical College of Huazhong University of Science and Technology, China. Informed consent was acquired from the participants or participants’ guardians. The patient’s information was confidential. An ID was given to each participant. There were no real names, initials, or disclose information that might identify a particular person. All procedures performed in studies involving human participants were in accordance with the 1964 Helsinki declaration and its later amendments^46,47^.

#### Subjects

Our study included 602 ASD patients and 604 healthy controls. The ASD patients were recruited from the Maternal and Child Care Service Centre in Shenzhen city, Zhuhai city and Luohu district in China, Wuhan Mental Health Center in China and Special Children’s Education Agency in Suzhou, Guangzhou, Wuhan and Tianjin in China between July 2010 and July 2018. ASD patients were diagnosed by professional neurologists based on the Diagnostic and Statistical Manual of Mental Disorders Fourth Edition before May 2013 or version 5 for cases diagnosed thereafter. The controls were recruited from the children who had a physical examination in the Maternal and Child Care Service Centre in Shenzhen city and from the primary schools in Wuhan. The control children were healthy population without ASD, attention-deficit/hyperactivity disorder, mental retardation or other neurodevelopmental disorders, and they were matched with ASD patients in gender^46,47^.

##### Genotyping of Candidate SNPs

Genomic DNA was extracted from oral swabs sample using TIANamp Swab DNA Kit DP080714 (Tiangen, Beijing, China) and from blood samples using RelaxGene Blood DNA System (Tiangen, Beijing, China) by reference to the manufacturer’s instructions. The type of samples depended on the choice of parents and children. DNA concentration and optical density were tested by a NanoDrop 1000 spectrophotometer (Thermo Fisher Scientific, Waltham, MA). Genotyping was performed at the BIO MIAOBIOLOGICAL Corporation (Beijing, China) with the SequenomMassARRAY platform (San Diego, CA) according to the manufacturer’s protocol. The MassARRAY Assay Designer software (v3.1) was used to design PCR primers and termination mixes for multiplexed assays. The mass of extended primer was determined using a MALDI-TOF mass spectrometer and we analyzed the resulting genotype spectra using Mass ARRAY Type4.0 software^46,47^.

### Statistical analysis

The ggplot2 package (http://ggplot2.org/) in R (v3.2.5) was used to plot the spatial-temporal expression patterns of *ITIH3* and the association between SNPs and the expression of *ITIH3*. SPSS software v22.0 was used for statistical analyses in experiment of Chinese population. The Hardy-Weinberg equilibrium (HWE) for genotypes was analyzed by Goodness-of-fit χ^2^ test in the healthy controls. Odds ratios (OR) and 95% confidence intervals (95% CI) were adopted to assess the relative risk conferred by a possibly risk allele and genotype. The statistical power to detect the effects of the SNPs was calculated by Power v3.0.0. For example, for SNPs with minor allele frequency (MAF) of 0.4709, and the prevalence of ASD in China was 2.00%, the power of the sample size to detect an OR of 1.50 was 93.6%. All *P* values were two-tailed with a statistical significant level set at 0.05^46,47^.

Multiple genetic models are used to estimate the associations between ASD susceptibility and candidate SNPs. The most commonly used five models include addictive, multiplicative, dominant, recessive, and over-dominant models^48,49^. A SNP with two alleles consists of a major allele (M) and a minor allele (m). The genotype can be a major allele homozygote (MM), a heterozygote (Mm) or a minor allele homozygote (mm). We chose the best model for each SNP following a new method proposed by Horita *et al*.^50^ to better control type I error. Generally, this mainly includes 4 steps: Step 1, OR1_ori_ and OR2_ori_ are estimated from the original number of subjects observed (OR1_ori_ = odd_Mm_/odd_MM_; OR2_ori_ = odd_mm_/odd_Mm_, odd = the number of cases/the number of controls carrying corresponding genotypes). Step 2, best model is selected based on OR1_ori_ and OR2_ori_ by plotting (OR1_ori_, OR2_ori_) on the log-scale OR1-OR2 plane. Step 3, OR_step3_ is obtained from unconditioned logistic regression under selected models. Step 4, OR1_mod_ and OR2_mod_ are calculated based on the optimal genetic models for each SNP. The details for this calculation can be found in Horita *et al*.^50,51^.

## Supplementary information

Supplementary Information.

Supplementary data.

## Data Availability

The data in the genotyping experiment in the Chinese Han population are available from the corresponding author on reasonable request. Other parts of data in this article are available in the Supplementary data.
