# Supplementary material for: Integrative analyses indicate an association between ITIH3 polymorphisms with autism spectrum disorder
Source: Sci Rep. 2020 Mar 23;10:5223. doi: 10.1038/s41598-020-62189-3 (PMC7089985; doi:10.1038/s41598-020-62189-3)
Supplement: Supplementary file 1 — Supplementary Information. [file 41598_2020_62189_MOESM1_ESM.pdf]

Integrative analyses indicate an association between *ITIH3* polymorphisms with autism spectrum disorder

Xinyan Xie[1#], Heng Meng[1#], Hao Wu[1], Fang Hou[2], Yanlin Chen[2], Yu Zhou[1], Qi Xue[1], Jiajia Zhang[3], Jianhua Gong[2], Li Li[2*] and Ranran Song[1*]

1Department of Maternal and Child Health and MOE Key Lab of Environment and Health, School of Public Health, Tongji Medical College, Huazhong University of Science and Technology, Wuhan 430030, China

2Maternity and Children Health Care Hospital of Luohu District, Shenzhen 518019, China

3Department of Epidemiology and Biostatistics, Arnold School of Public Health, University of South Carolina, Columbia, SC 29208, USA

[¶] These authors should be considered joint first author.

[*] These authors should be considered joint senior author.

Table S1 Basic information of SNPs in study

Gene	SNP	MA	MAF[†]	MAF[‡]	MAF[§]	*P*[¶]	Power(%)
ITIH3	rs2535629	A	0.3849	0.4709	0.4567	0.620	93.6
ITIH3	rs3617	A	0.4333	0.4854	0.4856	0.912	94.0

Abbreviations: MA, minor allele; MAF, minor allele frequency.

[†] The minimum allele frequency of the SNPs in the control group.

[‡] The minimum allele frequency of the SNPs in the 1000 Genomes Project in the Han Chinese in Beijing, China.

[§] The minimum allele frequency of the SNPs in the 1000 Genomes Project in the Japanese in Tokyo, Japan.

[¶] Hardy-Weinberg equilibrium test.

Table S2 Association between SNPs and ASD

Gene	SNP	Ref	Case(n)			Control(n)			$OR1_{ori}/OR1_{mod}$	$OR2_{ori}/OR2_{mod}$	Optimal model	OR_{step3}(95%CI) [†]	*P*[†]
			MM	Mm	mm	MM	Mm	mm					
ITIH3	rs2535629	A	67	307	222	91	276	228	1.511/0.746	0.875/1.340	Ov	0.746(0.578-0.963)	**0.024**
ITIH3	rs3617	A	92	307	197	112	296	192	1.263/0.973	0.989/1	Do	0.973(0.696-1.360)	0.872

Note: Abbreviations: Ref, reference allele/major allele; MM, homozygote of major allele; Mm, heterozygote; mm, homozygote of minor allele; Re, recessive model; Do, dominant model; Ov, over-dominant model; OR, odds ratio; CI, confidence interval. n is the number of cases and controls in each group. OR_{step3} was obtained from unconditioned logistic regression under selected models. [†] The OR and *P* values were corrected by gender, age, birth weight and child-born diseases. The significant level was corrected by Bonferroni method (α' = 0.05/2 = 0.025).The significant results were in bold.

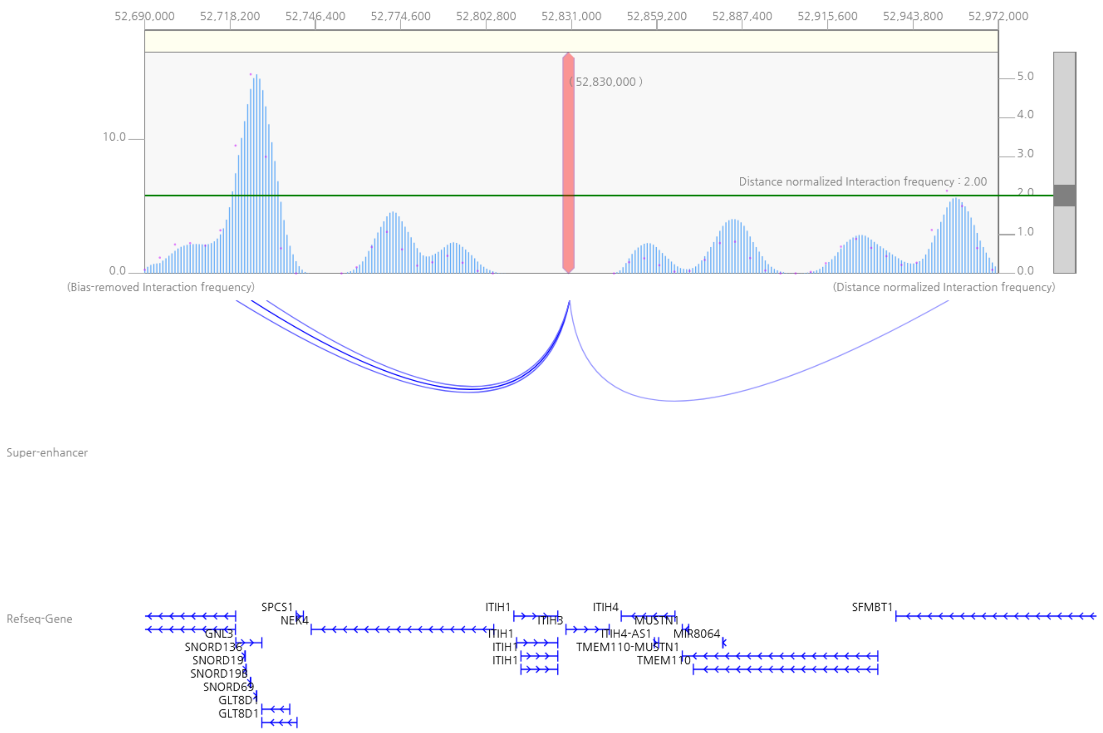

Figure S1 The SNP rs2535629 involved chromatin interactions in hippocampus tissues at chr3:52,690,000-52,972,000. The up panel showing One-to-all interactions with rs2535629; the X-axis showing the hg19 coordinates of the genomic region of interest; Y-axes on the left and the right indicate bias-removed interaction frequency (blue bar graph) and distance-normalized interaction frequency (magenta dots), respectively; Green line indicates the cut-off for distance-normalized interaction frequency. The middle panel uses arc-representation of significant interaction for the given cut-off value defined by the green line above. The bottom panel showing any Super-enhancer and Refseq genes mapped to the region of interest.

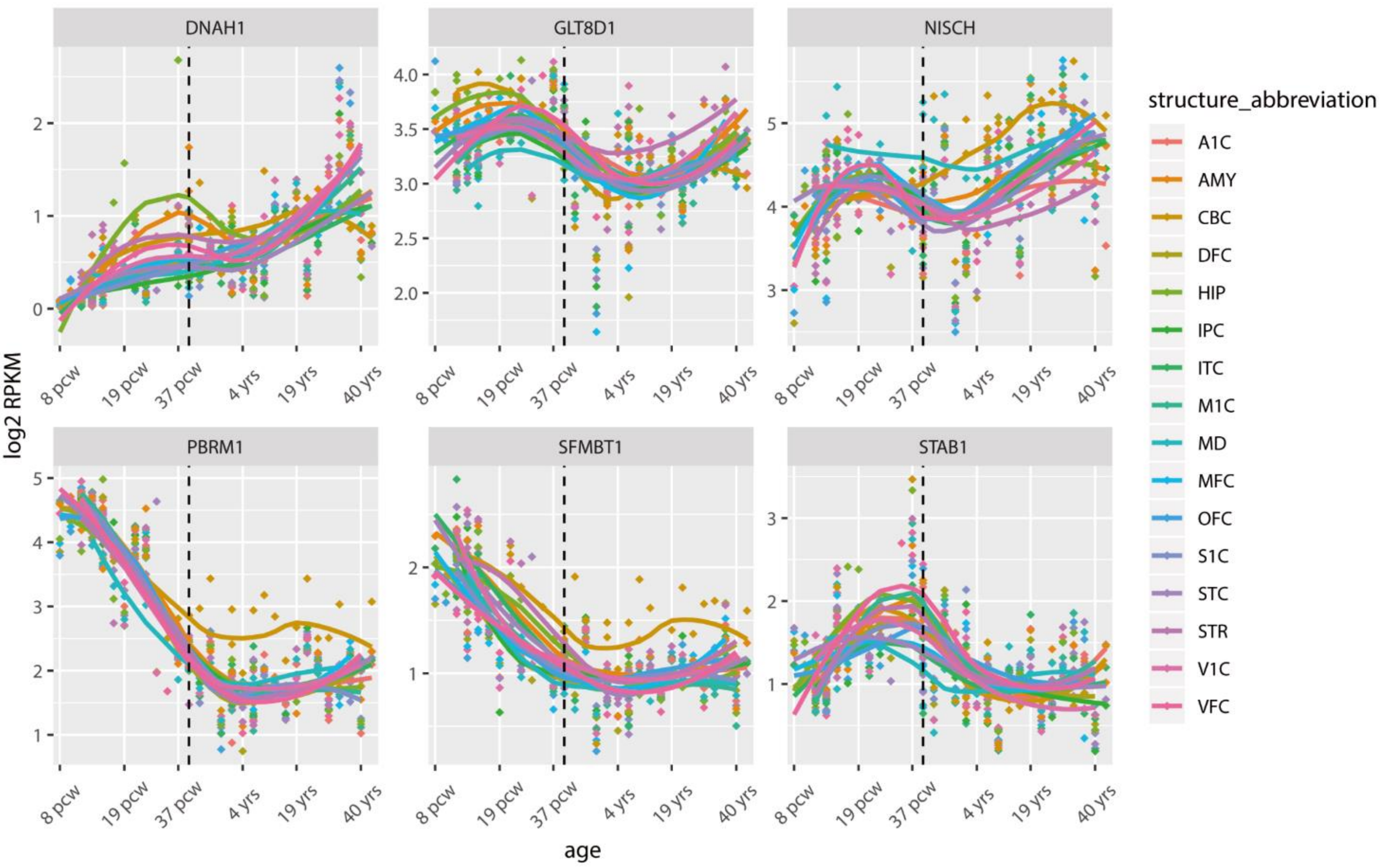

Figure S2 The other six SNP *cis*-regulatory effect genes expression in brain subtissues across brain development (with RNA-seq RPKM values from the BrainSpan Atlas v.10; shown from 8 postconception weeks (pcw) to 40 years of age (yrs)). Birth is indicated by a vertical gray dashed line. The brain structures are primary auditory cortex (core) (A1C), amygdaloid complex (AMY), cerebellar cortex (CBC), dorsolateral prefrontal cortex (DFC), hippocampus (HIP), posteroventral (inferior) parietal cortex (IPC), inferolateral temporal cortex (area TEv, area 20) (ITC), primary motor cortex (area M1, area 4) (M1C), mediodorsal nucleus of thalamus (MD), anterior (rostral) cingulate (medial prefrontal) cortex (MFC), orbital frontal cortex (OFC), primary somatosensory cortex (area S1, areas 3,1,2) (S1C), posterior (caudal) superior temporal cortex (area 22c) (STC), striatum (STR), primary visual cortex (striate cortex, area V1/17) (V1C), and ventrolateral prefrontal cortex (VFC).
